# Small fibre pathology, small fibre symptoms and pain in fibromyalgia syndrome

**DOI:** 10.1038/s41598-024-54365-6

**Published:** 2024-02-16

**Authors:** Anne Marshall, Leandros Rapteas, Jamie Burgess, David Riley, Matthew Anson, Kohei Matsumoto, Amanda Bennett, Stephen Kaye, Andrew Marshall, James Dunham, Nicholas Fallon, Sizheng S. Zhao, Anne Pritchard, Nicola Goodson, Rayaz A. Malik, Andreas Goebel, Bernhard Frank, Uazman Alam

**Affiliations:** 1 Institute of Life Course and Medical Sciences, University of Liverpool, Liverpool, UK; 2 Liverpool University Hospitals NHS Foundation Trust, Aintree Hospital, Liverpool, UK; 3 The Walton Centre NHS Foundation Trust, Liverpool, UK; 4 School of Physiology, Pharmacology and Neuroscience, University of Bristol, Bristol, UK; 5 Institute of Population Health, University of Liverpool, Liverpool, UK; 6 Faculty of Biological Medicine and Health, Centre for Epidemiology Versus Arthritis, University of Manchester, Manchester, UK; 7 Fibromates, North West Fibromyalgia Support Group, Liverpool, UK; 8 Research Institute for Sport and Exercise Science, Liverpool John Moores University, Liverpool, UK; 9 Research Division, Weill Cornell Medicine-Qatar, Qatar Foundation, 24144 Doha, Qatar

**Keywords:** Medical research, Neurology, Rheumatology

## Abstract

A proportion of people with fibromyalgia demonstrate small fibre pathology (SFP). However, it is unclear how SFP directly relates to pain phenomenology. Thirty-three individuals with FMS and ten healthy volunteers underwent assessment of SFP and sensory phenotyping using corneal confocal microscopy, validated questionnaires and quantitative sensory testing (QST). Corneal nerve fibre length was used to stratify participants with fibromyalgia into with SFP [SFP+] and without SFP [SFP−]. SFP was detected in 50% of the fibromyalgia cohort. Current pain score and QST parameters did not differ between SFP+ and SFP−. Mechanical pain sensitivity (MPS) demonstrated a significant gain-of-function in the SFP− cohort compared to healthy-volunteers (p = 0.014, F = 4.806, η^2^ = 0.22). Further stratification revealed a cohort without structural SFP but with symptoms compatible with small fibre neuropathy symptoms and a significant gain in function in MPS (p = 0.020 Chi-square). Additionally, this cohort reported higher scores for both depression (p = 0.039, H = 8.483, η^2^ = 0.312) and anxiety (p = 0.022, F = 3.587, η^2^ = 0.293). This study confirms that SFP is present in a proportion of people with fibromyalgia. We also show that in a proportion of people with fibromyalgia, small fibre neuropathy symptoms are present in the absence of structural SFP. Greater mechanical pain sensitivity, depression and anxiety are seen in these individuals.

## Introduction

Fibromyalgia syndrome (FMS) is a chronic pain condition characterized by widespread pain of unclear aetiology. The estimated prevalence of FMS differs according to the diagnostic criteria used, and ranges from 2 to 6% in the general population worldwide^1^. Pain in FMS is frequently accompanied by fatigue, sleep disturbance, cognitive dysfunction, and significant functional disability^2^. Depression and anxiety are frequent comorbidities, with approximately two-thirds taking anti-depressant medication^3^.

FMS is widely considered to originate in or be amplified by the central nervous system (CNS)^4,5^. Indeed, multiple chronic pain studies have reported alterations in CNS mechanisms, including impairment of diffuse noxious inhibitory control, measured using conditioned pain modulation (CPM)^6^. However, there is increasing evidence of the potential role of peripheral mechanisms in the pathogenesis of FMS symptoms, with a subset of patients developing small fibre deficits. Combined with possible features of peripheral neuropathic pain including burning, paraesthesia, hyperalgesia and allodynia^7,8^, the origin of FMS pain may in a proportion of people stem from the peripheral nervous system. A systematic review and meta-analysis indicated small nerve fibre loss occurring in approximately 50% of patients with FMS^9^. As well structural alterations, abnormal spontaneous activity and/or sensitisation of nociceptive c-fibres have been identified in patients with FMS, suggestive of involvement in pain generation/maintenance^10^. Additionally, it has been argued that abnormalities in pain-evoked potentials and quantitative sensory testing (QST) parameters suggest a potential peripheral mechanism^7^, although this remains unclear.

Small nerve fibres may be evaluated through symptoms, quantitative sensory testing, and skin biopsy. However, the development of corneal confocal microscopy (CCM), a rapid, non-invasive ophthalmic imaging technique, has enabled quantification of corneal nerve fibres and detection of small fibre loss^11,12^. CCM can detect subtle changes in corneal nerve fibre pathology^13^ and is extensively utilised as a surrogate marker of small fibre neuropathy and peripheral neuropathy-related disorders in research^14^. CCM has demonstrated comparable diagnostic utility to intraepidermal nerve fibre density (IENFD)^8,15^, although some studies describe conflicting results^16^. Reduction in corneal nerve parameters including fibre density (CNFD)^8,17,18^, fibre length (CNFL)^8,18^ and branch density (CNBD)^18^ have all been demonstrated in FMS. Conversely, there are a paucity of research evaluating the association between abnormalities in corneal nerve fibres, QST and patient reported symptoms or treatment outcomes. Indeed, the mechanisms that lead to small fibre pathology and dysfunction and whether FMS should be considered partly as a neuropathic pain disorder, remain a matter of debate^19,20^.

We aimed to evaluate whether there are distinct FMS phenotypes based on the presence or absence of SFP and small nerve fibre symptoms and subsequently detail the associated sensory phenotypes through an evaluation of QST.

## Results

Abbreviation section details all abbreviations used within the this section.

Data from 30 patients with FMS were included. Patient characteristics are detailed in Table 1. Twenty-eight (of 30) participants with FMS were female reflective of the general demographics of FMS. The mean age in FMS participants was 44 ± 15 years with a duration since diagnosis of 6 years. Fibromyalgia symptoms were present for an average of 5.5 years prior to diagnosis. Average BMI was in the overweight range (29.0 ± 8.0 kg/m^2^), and mean HbA1c and triglyceride level was at the upper range of normal. Median pain (VAS, 76/100; PainDetect, 27/38), depression (6/10) and anxiety (7/10) scores were high, as were neuropathy symptoms scores (SFNSL, 50/84; NSP, 19/33). Mean values for CNFD, CNBD and CNFL fell within normal range, although data from individual participants (1/30 for CNFD, 2/30 for CNBD, 15/30 for CNFL) did fall below the normative range^21^. An abnormal reduction in CNFL was observed in 50% of participants with FMS and were thus stratified into FMS with (SFP+) (n = 15) and without (SFP−) (n = 15) SFP, based on below and within normative CNFL, respectively (see “Methods” section).

**Table 1:** Patient characteristics.

Fibromyalgia participants	30
Gender (F/M)	28/2
Age (years)	44 ± 15
Body mass index (BMI) (kg/m^2^)	29.0 ± 8.0
Blood pressure (systolic/diastolic) (mm/Hg)	126/72 ± 19/12
HbA1c (mmol/mol)	36.2 ± 4
Triglycerides (mmol/L)	1.7 ± 1.0
Time since diagnosis (years)	6.0 (1.8–9.3)
Duration of symptoms prior to diagnosis (years)	5.5 (2.8–10.0)
Corneal nerve fibre density (CNFD)	24.7 ± 5.7
Corneal nerve branch density (CNBD)	33.0 ± 13.0
Corneal nerve fibre length (CNFL)	14.7 ± 3.0
VAS current pain score	76 (62–86)
FIQR total (score out of 100)	77.3 (56.2–84.6)
FIQR depression (score out of 10)	6.0 (2.0–9.3)
FIQR anxiety (score out of 10)	7.0 (3.8–9.0)
Small fibre neuropathy screening list (SFNSL)	50 (44–60)
PainDetect total (out of 38)	27 (21–30)
Neuropathy symptom profile (NSP)	19 (14–24)

Demographic, anthropometric, metabolic, CCM, reported pain and neuropathy symptom parameters in patients with FMS. Parametric data are mean ± standard deviation. Non-parametric data are median ± interquartile range. SFNSL, < 11 = SFN less likely, 11–48 = probable/likey SFN, > 48 = symptoms likely of SFN; PainDetect, > 18 likely neuropathic pain; NSP, 0 = no, 1–9 = mild, 10–18 = moderate, and 19–33 = severe polyneuropathy. *VAS* visual analogue score; *FIQR* fibromyalgia impact questionnaire (revised).

Group characteristics are detailed in Table 2. There was no difference in mean age, BMI, systolic or diastolic blood pressure, HbA1c and triglycerides across the three groups. There was no difference between time to diagnosis or symptoms prior to diagnosis between the two FMS cohorts. Based on the International Diabetes Federation (IDF) criteria^22^, two patients with FMS met the guidelines for metabolic syndrome based on waist circumference (≥ 80 cm for women 31) plus elevated triglycerides and blood pressure. No control subjects met these criteria.

**Table 2:** Group characteristics.

	FMS without SFP (SFP−) (n = 15)	FMS with SFP (SFP+) (n = 15)	Healthy volunteers (n = 10)	One-way ANOVA	Effect size eta squared
Demographics					
Gender (F:M)	14:1	14:1	9:1		
Age (years)	42.35 ± 14.8	45.4 ± 15.0	42.6 ± 11.8	F = 0.191 p = 0.827	
Duration (years)	6.5 ± 4.5	6.2 ± 5.3	–	F = 1.415 p = 0.895	
Symptoms prior diagnosis (years)	8.7 ± 9.5	9.5 ± 13	–	F = 1.865 p = 0.844	
Anthropometrics					
BMI (kg/m^2^)	27.2 ± 7.3	30.9 ± 8.4	24.7 ± 12.9	F = 1.158 p = 0.326	
Systolic BP (mmHg)	118 ± 12	132 ± 22	121 ± 12	F = 2.415 p = 0.107	
Diastolic BP (mmHg)	73 ± 8	79 ± 14	77 ± 7	F = 0.785 p = 0.466	
Biochemistry					
HbA1c (mmol/mol)	34.9 ± 4.0	37.5 ± 3.9	36.3 ± 5.4	F = 1.321 p = 0.281	
Triglycerides (mmol/L)	1.6 ± 0.8	1.8 ± 1.1	1.0 ± 0.4	F = 1.400 p = 0.263	
Corneal confocal microscopy					
CNFD (number of major nerves/mm^2^)	29.6 ± 2.7	19.9 ± 3.2	26.8 ± 4.0	F = 36.19** p ≤ 0.001**^**+++**^**^^^**	**0.66**
CNBD (number of nerve branches/mm^2^)	42.6 ± 10.8	23.5 ± 6.7	43.2 ± 15.6	F = 14.60** p ≤ 0.001**^**+++**^**^^^**	**0.44**
CNFL (length of nerves/mm^2^)	17.1 ± 1.5	12.3 ± 2.0	16.0 ± 3.0	F = 20.17** p ≤ 0.001**^**+++**^**^^^**	**0.52**
Conditioned pain modulation					
CPM	0.35 ± 0.63	0.79 ± 0.63	1.70 ± 2.44	F = 2.223 = 0.131	
Participant reported pain and questionnaires					
Median (interquartile range)					
VAS (current pain score 0–100)	80.8 (52.9–90.2)	73.5 (62.3–80.1)	0 (0–7.7)	H = 21.09 p ≤ 0.001***^^^	**0.46**
FIQR (total score)	80.2 (54.2–100)	76.0 (64.7–83.0)	0 (0–4.5)	H = 22.2 p ≤ 0.001***^^^	**0.47**
SFNSL (total score)	52.5 (41.3–63.5)	50.0 (44.0–55.0)	2.0 (0–3.0)	H = 22.7 p ≤ 0.001***^^^	**0.51**
PainDetect (total score)	26.8 (18.0–28.8)	28.0 (21.0–30)	0 (0–0.8)	H = 18.5 p ≤ 0.001***^^^	**0.50**
Quantitative sensory testing z-scores (the testing site for QST was the dorsum of the hand)					
Mean ± SD					
CDT (z-score)	− 0.51 ± 0.90	− 1.02 ± 0.99	− 0.24 ± 0.84	F = 2.356 p = 0.109	
WDT (z-score)	− 0.91 ± 1.24	− 0.79 ± 0.74	− 0.11 ± 0.85	F = 2.211 p = 0.124	
TSL (z-score)	− 0.60 ± 1.06	− 0.87 ± 0.97	0.00 ± 0.76	F = 2.465 p = 0.099	
CPT (z-score)	0.47 ± 1.21	0.41 ± 1.15	− 0.55 ± 0.96	F = 2.883 p = 0.069	
HPT (z-score)	0.31 ± 1.48	0.65 ± 1.34	− 0.61 ± 1.01	F = 2.804 p = 0.074	
PPT (z-score)	4.11 ± 3.61	4.21 ± 2.52	0.36 ± 1.89	F = 5.993 p = 0.006*^^	**0.25**
MDT (z-score)	− 1.34 ± 2.23	− 0.70 ± 1.24	0.22 ± 1.60	F = 2.339 p = 0.111	
MPT (z-score)	0.65 ± 1.21	0.95 ± 1.27	1.29 ± 1.57	F = 0.659 p = 0.523	
MPS (z-score)	1.91 ± 1.86	0.86 ± 1.36	− 0.03 ± 0.92	F = 4.806 p = 0.014*	**0.22**
WUR (z-score)	0.23 ± 1.29	0.26 ± 1.04	− 0.64 ± 0.53	F = 2.243 p = 0.121	
Median (interquartile range)					
VDT (z-score)	0.41 (− 0.44 to 0.65)	0.56 (0.41–0.61)	0.5 (0.4–0.6)	H = 0.611 p = 0.737	
DMA	0 (0 to 3.42)	0 (0–0)	0 (0–0)	H = 2.006 p = 0.367	
PHS	0 (0 to 0)	0 (0–0)	0 (0–0)	H = 0.130 p = 0.937	

Demographics, anthropometric, metabolic, corneal confocal microscopy, reported pain and quantitative sensory testing parameters in FMS patients, with and without SFP and control subjects. Mean ± standard deviation and test statistic F reported for parametric data. Median ± interquartile range and test statistic H reported for non-parametric data. One-way ANOVA. In parentheses, Tukey’s multiple comparisons test for parametric data and Dunn’s multiple comparisons test for non-parametric data; ^+++^p ≤ 0.001 between FMS SFP− and FMS SFP+, ^^p ≤ 0.01 and ^^^p ≤ 0.001 between FMS SFP+ and controls, *p ≤ 0.05 and ***p ≤ 0.001 between FMS SFP− and controls. Effect size (eta squared(η2)) was reported for values that reached the significance threshold set at < 0.05.

Significant values are in bold.

### Quantitative sensory testing

**Figure 1:**
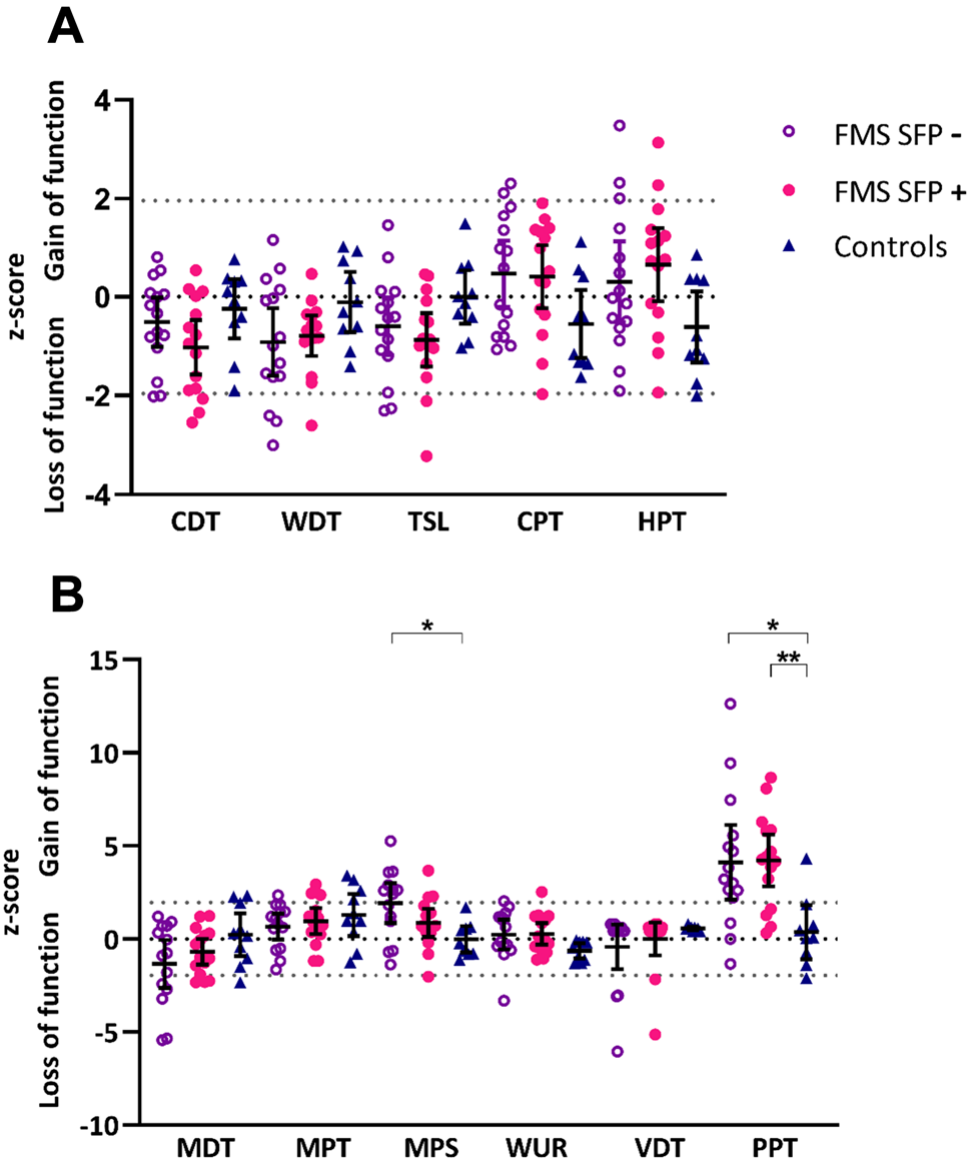
Sensory profiles. (**A**) Individual and group mean ± SD of z-scores for thermal quantitative sensory testing parameters in patients with FMS without (purple hollow circles) and with (pink circles) SFP based on CCM assessment and control subjects (dark blue triangles). (**B**) Individual and group mean ± SD of z-scores for mechanical quantitative sensory testing parameters in patients with FMS without (purple hollow circles) and with (pink circles) SFP based on CCM assessment and control subjects (dark blue triangles). One-way ANOVA and Tukey’s multiple comparison test for parametric data and Kruskal–Wallis test and Dunn’s multiple comparison test for non-parametric data; *p ≤ 0.05. *CDT* cold detection threshold, *WDT* warm detection threshold, *TSL* thermal sensory limen, *CPT* cold pain threshold, *HPT* heat pain threshold, *MDT* mechanical detection threshold, *VDT* vibration detection threshold, *MPT* mechanical pain threshold, *MPS* mechanical pain sensitivity, *WUR* wind-up ratio, *PPT* pressure pain threshold.

Individual z-scores for QST parameters are summarized in Fig. 1. Mean z-score for all parameters fell within the normative range of DFNS control data with the exception of PPT in both patient groups, which demonstrated a significant gain of function. There was no significant difference in any QST parameter between participants with FMS with and without small fibre pathology. However, patients in the SFP− patient cohort had a significant gain of function in mechanical pain sensitivity (p = 0.014, F = 4.806, η^2^ = 0.22) compared to healthy volunteers, indicative of significantly increased mechanical pain scores. As expected, participants with FMS, both SFP+ and SFP−, demonstrated a significant gain in function in PPT compared to healthy volunteers, indicative of significantly reduced pressure pain thresholds. There was no significant difference in conditioned pain modulation either between all FMS participants and healthy volunteers or between any of the sub-groups.

### Questionnaires

**Figure 2:**
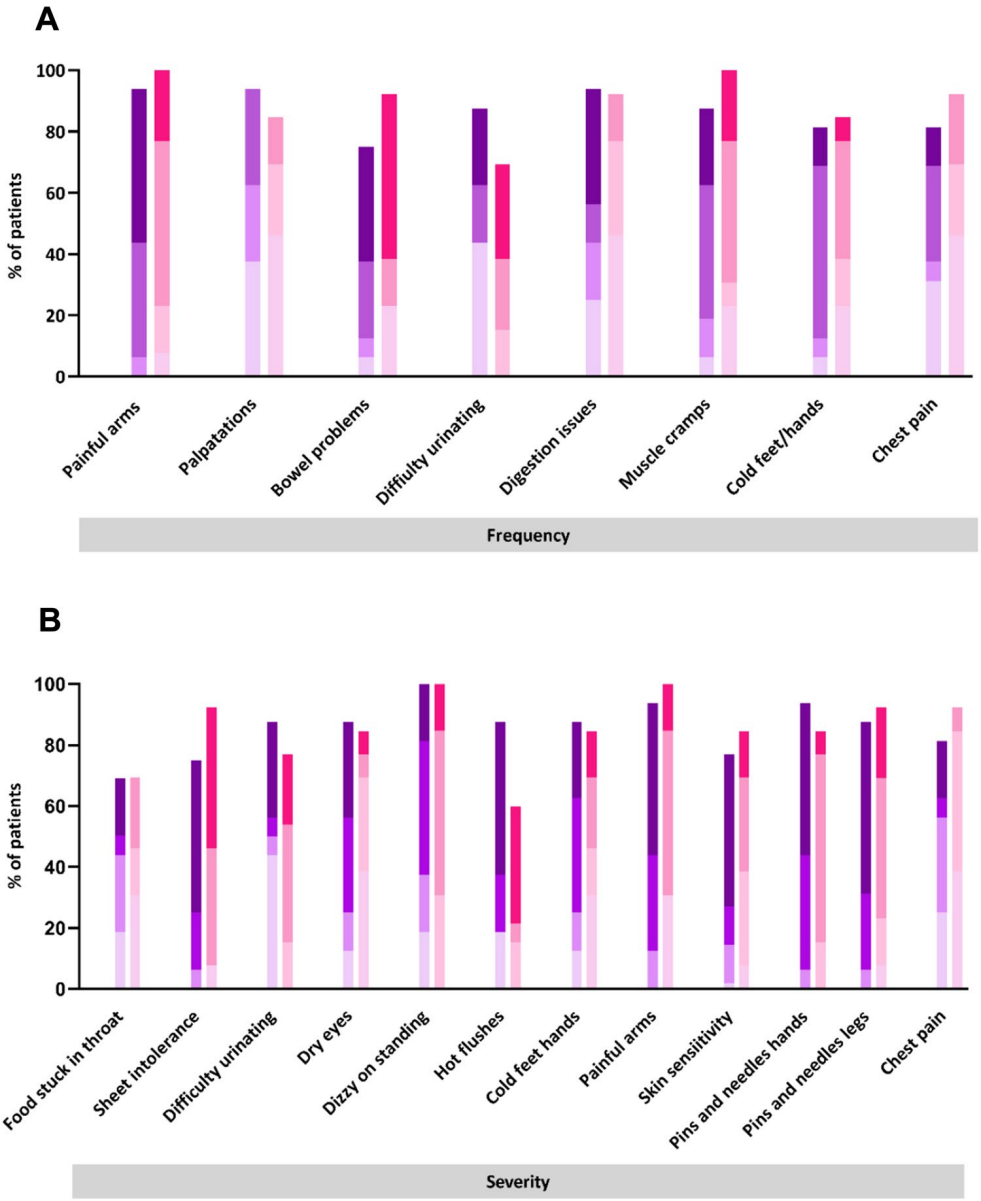
Small fibre neuropathy screening list complaints in patients with fibromyalgia. (**A**) Small fibre neuropathy symptom frequency in FMS patients without (purple) and with (pink) SFP on CCM assessment. (**B**) Small fibre neuropathy symptom severity in FMS patients without (purple) and with (pink) SFP on CCM assessment. Bar height represents the overall percentage of patients experiencing the complaint. Bar shading represents the proportion of patients experiencing the complaint sometimes/always/often/always and with severity slightly/variably/moderately/seriously. The darkness of shading increases with increasing frequency and severity of symptoms.

The total score on all 5 questionnaires (FIQR, SFNSL, PainDetect, NSP and McGill VAS) were higher in both FMS cohorts compared to healthy volunteers (p > 0.001). Eight participants with SFP, and 9 participants without SFP based on CNFL, had symptoms compatible with small fibre neuropathy symptoms on the SFNSL questionnaire. Twenty-six participants (14/15 with and 12/15 without SFP based on CNFL) had a score on PainDetect compatible with neuropathic pain (> 18), demonstrating that symptoms compatible with neuropathic pain are present in individuals with FMS, with and without SFP. There were no differences in FIQR, PainDetect, McGill VAS or NSP total score, or in any individual SFNSL symptom frequency or severity, between the SFP+ and SFP− cohorts (Fig. 2).

### Corneal confocal microscopy

**Figure 3:**
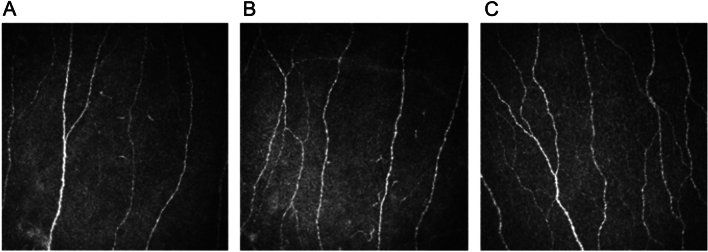
CCM raw images. Representative CCM images from patients with FMS (**A**) with SFP and (**B**) without SFP compared to (**C**) control subject; (380 × 380 pixels with an area of 400 × 400 mm^2^).

Representative CCM images are shown in Fig. 3.

**Figure 4:**
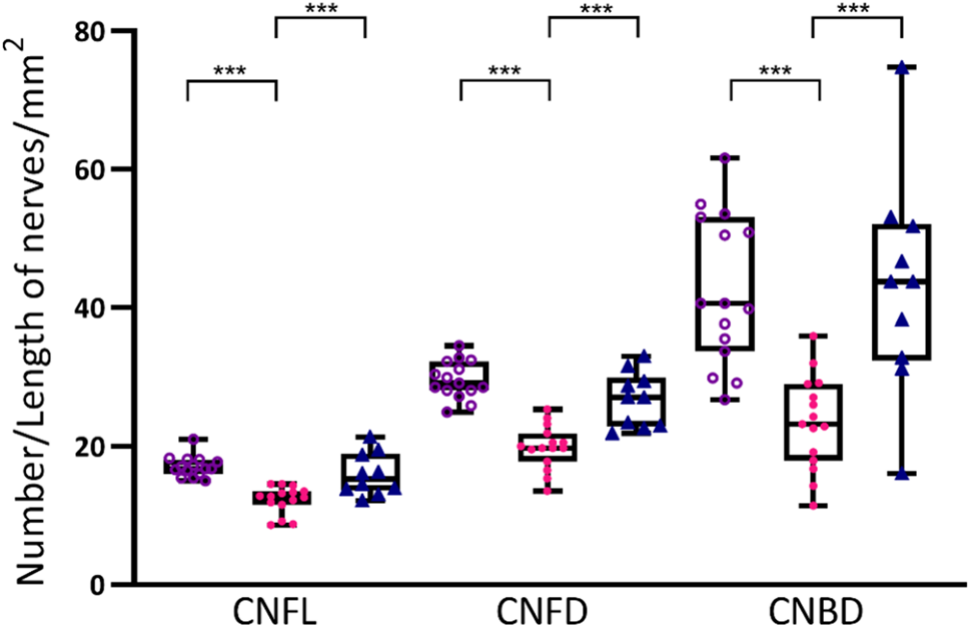
Corneal confocal microscopy parameters. Individual and group mean of CNFL, CNFD, and CNBD in patients with FMS without (purple hollow circles) and with (pink circles) SFP and control subjects (dark blue triangles). One-way ANOVA and Tukey’s multiple comparison test ***p ≤ 0.001. *CNFL* corneal nerve fibre length, *CNFD* corneal nerve fibre density, *CNBD* corneal nerve branch density.

Patients were delineated based on CNFL, therefore as expected patients with SFP+ had reduced CNFL compared to participants within the SFP− cohort and healthy volunteers. Patients in the SFP+ cohort additionally had reduced CNFD and CNBD compared to participants within the SFP− cohort and healthy volunteers (p < 0.001). There were no significant differences in CNFL, CNFD, or CNBD between the SFP− and control group (Fig. 4).

Our results based on stratification indicate that 9 out of 15 participants with FMS who have no evidence of structural small fibre pathology on CCM, have scores which meet the criteria for small fibre neuropathy symptoms on the SFNSL questionnaire. Additionally, 7 out of 15 participants with FMS who do have evidence of structural small fibre pathology, do not report scores of symptoms compatible with small fibre neuropathy on SFNSL questionnaire.

**Figure 5:**
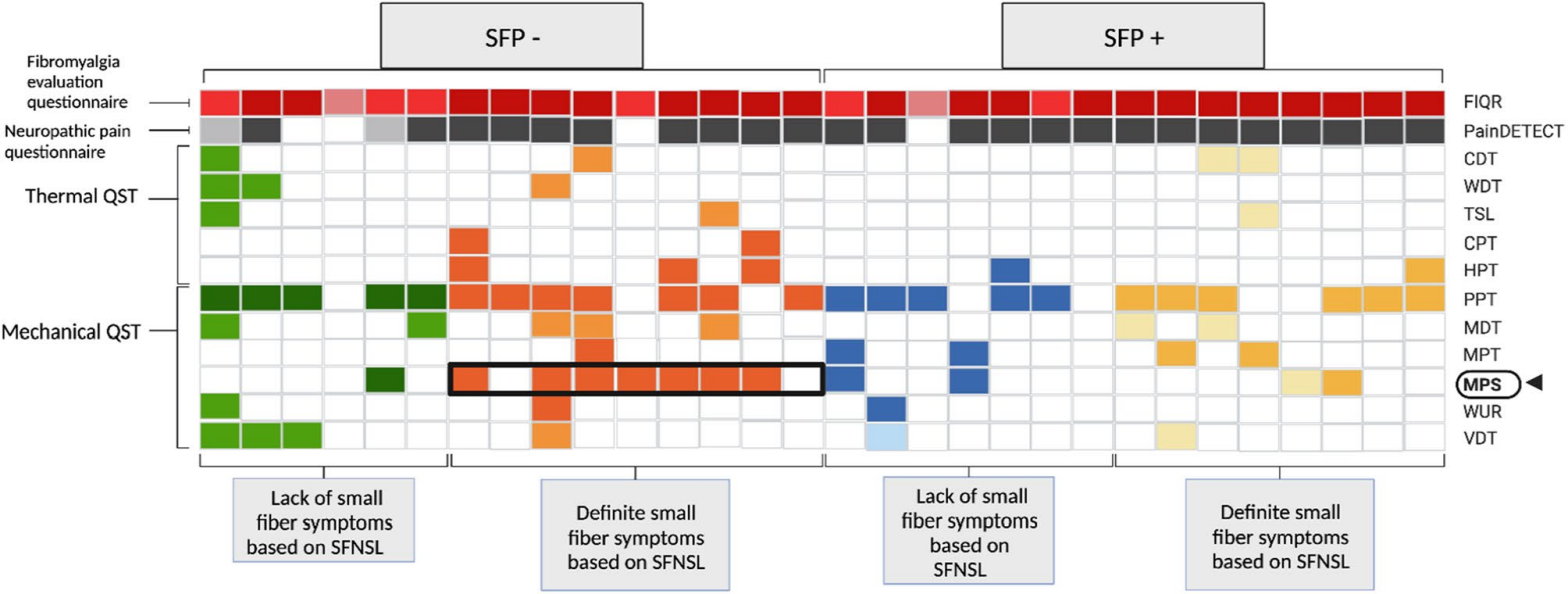
Individual patient phenotypes. 4 patient groups based on CNFL (SFP−/SFP+) and Small Fibre Neuropathy Screening List (SFNSL−/SFNSL+). Each column represents results from a single patient: normal (white) and abnormal (coloured). QST, gain of function is depicted with darker shade and loss of function is represented with lighter shade. PainDetect, negative (white) unclear (light grey) positive neuropathic pain (dark grey). FIQR, mild FMS symptoms (light shade), moderate FMS symptoms (mid shade), severe FMS symptoms (dark shade).

To further investigate whether there is a relationship between structural small fibre alterations on CCM and reported small fibre neuropathy symptoms, we further subdivided the SPF+ and SPF− patient cohorts into those without small fibre neuropathy symptoms and those with symptoms compatible with small fibre neuropathy based on SFNSL score (SFNSL−/SFNSL+ respectively) (Fig. 5).

**Figure 6:**
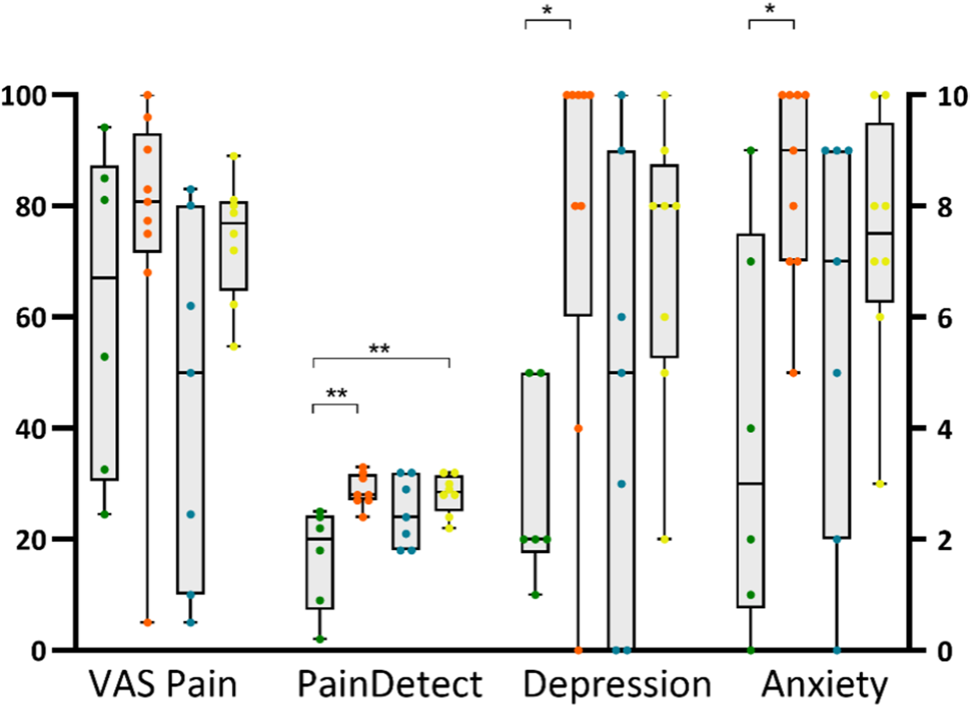
Pain, depression and anxiety. Individual current VAS pain, total PainDetect, FIQR depression and FIQR anxiety scores in patients with FMS: SFP−/SFNSL− (green circles); SFP−/SFNSL+ (orange circles); SFP+/SFNSL− (blue circles); SFP+/SFNSL+ (yellow circles). The centre line denotes the median value. One-way ANOVA and Tukey’s multiple comparison test for parametric data and Kruskal–Wallis test and Dunn’s multiple comparison test for non-parametric data; *p ≤ 0.05, **p ≤ 0.01.

The cohort of patients without SFP on CCM but with symptoms compatible with small fibre symptoms on SFNSL (SFP−/SFNSL+) had significantly more patients with a gain in function in MPS (7/9) compared to all other cohorts (Chi-square p = 0.020). These patients also reported significantly higher scores for depression (p = 0.039, H = 8.483, η^2^ = 0.312), anxiety (p = 0.022, F = 3.587, η^2^ = 0.293) and PainDetect total score (p = 0.003, H = 6.26, η^2^ = 0.429) compared to those without both structural SFP and small fibre neuropathy symptoms (SFP−/SFNSL−) (Fig. 6).

## Discussion

The primary aim of this study was to assess whether people with FMS who have evidence of small fibre pathology SFP have a distinct phenotype. Using CCM, we demonstrate an abnormal reduction in CNFL in half of the patients with FMS. Utilising CNFL to stratify participants with and without SFP, we also established additional corneal nerve pathology with reduced fibre density and branch density. Both groups had symptoms compatible with neuropathic pain and there was no significant difference in reported pain intensity or in any of the individual QST parameters between patients with FMS with and without SFP. However, compared to healthy volunteers, both FMS groups (SFP+/SFP−) demonstrated a significant gain-in-function in pressure pain threshold, in-keeping with the primary sensory characteristic described by patients with FMS.

However, of note, we demonstrate phenotypic differences in patients with FMS who display symptoms compatible with small fibre neuropathy symptoms but in the absence of corneal structural small fibre pathology. Mechanical pain sensitivity, a measure of pain ratings to pinprick stimuli, was also increased in the sub-group of patients without SFP pathology on CCM but with symptoms compatible with small fibre symptoms (SFP−/SFNSL+).

Our QST findings are in-keeping with those of previous FMS studies, with the majority of z-scores falling within the normative range of DFNS control data^23^. Similar to previous studies, our initial group wise comparison between FMS patients with and without alterations in corneal morphology suggests a minimal impact of SFP on somatosensory signs and symptoms^7,24–26^. However, more detailed analysis of our data, further stratifying based on reported small fibre symptoms, identified a distinct sensory phenotype where a gain of function in MPS is dominant. These findings lend support to the presence of a sub-group of patients with amplification along the mechanical pain pathway, with symptoms indicative of small fibre neuropathy in the absence of structural small fibre alterations. Previous investigations in individuals with FMS have also shown the presence of mechanical hypersensitivity^24,26^. Multiple lines of evidence suggest that central sensitisation contributes to the sensory phenomena of mechanical hypersensitivity^27,28^. In keeping with a role for central sensitisation in individuals with FMS without small fibre pathology, a recent study by Van de Donk et al. demonstrated that the efficacy of pain relief from Tapentadol, a centrally acting opioid with noradrenaline re-uptake inhibition potentiating descending pain modulation was predicted by inefficient conditioned pain modulation (CPM) in the presence of normal corneal nerve fibre morphology^29^. Whilst our study did not identify any significant difference in CPM between FMS and healthy volunteers, the role of abnormal descending inhibition in FMS is unclear^30,31^. However, pain in FMS is likely to relate to a complex balance between peripheral inputs and central processing. Larger cohorts incorporating data relating to central mechanisms (e.g., fMRI and H-reflex rate dependent depression)^32^ or direct measurement of peripheral inputs (microneurography)^8^ may help determine whether this pain amplification is peripherally or centrally driven.

Our demonstration of increased levels of anxiety and depression in the cohort of participants without SFP but with mechanical amplification is of potential mechanistic importance, given the additional reporting of significantly increased scores on the PainDetect questionnaire. This raises the possibility of either greater pain intensity resulting in increased levels of anxiety and depression, or increased levels of anxiety and depression leading to pain catastrophising. This may represent alterations in serotonergic/noradrenergic system which may represent a similar patient cohort as described by Van de Donk et al.^29^. In addition, Ramirez et al. have demonstrated that, in individuals with FMS, the correlation observed between small fibre loss and small fibre neuropathy symptom burden, assessed using the Small-Fiber Symptom Survey questionnaire, is distorted in individuals with higher levels of anxiety and depression^33^. The possibility of structural and functional alterations in brain regions associated with depression, specific to this sub-group of individuals with FMS, needs further consideration.

The aetiology of SFP in FMS remains unknown. However, recent investigations by our group suggest that SFP in FMS may have an autoimmune aetiology. Mice treated with IgG from patients with FMS not only develop hypersensitivity to cold and mechanical stimulation but also display loss of intraepidermal nerve fibres^34^. Furthermore, these putative autoimmune mechanisms may directly cause peripheral nerve pathology and sensitisation, as both mouse and human dorsal root ganglia, specifically satellite glial cells, were labelled with FMS IgG^34^. Furthermore, recent data demonstrate that a subset of fibromyalgia patients have elevated levels of anti-satellite glial cell antibodies, and the antibodies are associated with more severe fibromyalgia symptoms^35^. Consequently, it has been hypothesised that targeting a reduction in IgG titres in patients with FMS may be effective in reducing symptoms burden^34^. Conversely it has been argued that SFP in FMS may result from CNS dysfunction^36^. Administration of l-trans-Pyrrolidine-2,4-dicarboxylic acid (PDC), an inhibitor of glutamate transport (thus increasing glutamate levels) injected bilaterally in the insular in an experimental rat model, results in a consistent increase in multimodal pain behaviours and a decrease in peripheral nerve fibres^36^. Patients with FMS display reduced grey matter density in the insula compared to control subjects^37^ and also show enhanced resting state connectivity between the insular cortex and default mode network, which correlates with spontaneous clinical pain intensity at the time of scanning^38^. Whilst these imaging findings were not related to peripheral nerve pathology, in a recent cross-sectional imaging study in 43 women with FMS, Aster et al.^39^ tested the hypothesis that reduced skin innervation in FMS is associated with specific CNS alterations. The subgroup with reduced skin innervation (n = 21 of 43) demonstrated hyperconnectivity between a number of brain regions including the inferior frontal gyrus, the angular gyrus and the posterior parietal gyrus^39^. Additional alterations were noted including lower volumes in bilateral pericalcarine cortex and lower fractional anisotropy in the left posterior limb of internal capsule and in the posterior thalamic radiation, both in the PNS group^39^.

We have also previously demonstrated that SFP occurs in ~ 50% of people with FMS demonstrated by CCM or intra-epidermal nerve density through skin biopsy^9^. However, other studies have demonstrated a differential effect of FMS with lower intra-epidermal nerves at the thigh versus the distal leg^4^. Although, this study did not assess intra-epidermal nerves, our future work will include skin biopsy analysis and brain imaging (structural/fMRI) and we suggest a similar multi-modal evaluation is incorporated in future mechanistic studies of FMS by other groups. Additionally, the clinical presentation of small fibre neuropathy and QST findings from other aetiologies e.g. cryptogenic, diabetes amyloidosis, etc. are distinct from the findings in this study, which does raise questions on the clinical significance of SFP in FMS. Histological data alone are considered insufficient^40^ and even with the addition of questionnaires with compatible symptoms, caution is required in the interpretation of SFP as definitive small fibre neuropathology. From the data in this study, it is uncertain whether established SFP in FMS plays a role in the symptomatology.

## Limitations and future research

Our initial data may reflect different underlying pathophysiological mechanisms with associated FMS phenotypes resulting from interactions between peripheral and central processes^7,41^. Although the CCM and QST findings are representative of previously reported FMS populations, our finding of a sub-group of patients with symptoms of small fibre neuropathy and mechanical hyperalgesia in the absence of small fibre pathology is from a relatively small sample size. Also, symptoms that are evaluated by SNFL are frequently present in patients with FMS without alterations of small fibre neuropathy. Further work, including a larger sample size, and the addition of cluster analysis is required to confirm these initial findings. The study is cross-sectional therefore, one limitation is that patient reported symptoms and pain scores were evaluated at the point of assessment and may be subject to some recall bias. Patients also continued to take pain medication which has not been systematically accounted for in these preliminary analyses and would be expected to impact on the pain ratings. The CPM paradigm using the contralateral site for the conditioning may introduce a segmental effect and potential associated bias. Also, we did not undertake QST on the trunk in which normative values are available. These additional data may have been helpful when considering typical pressure point areas. Future mechanistic studies should include structure and functional assessment of the entire neuroaxis and peripheral nervous system to delineate the underlying mechanisms in this complex syndrome, and to subsequently test differential responses to drugs (e.g., lidocaine infusion, esketamine infusions, pure opioids and mixed opioid like tapentadol) to predict a better response based on the presence or absence of SFP with other mechanisms.

In conclusion, we demonstrate that SFP is present in half of people with FMS. Additionally, we have described distinct phenotypes based on small nerve fibre structure and small fibre symptoms, including a subpopulation of participants with normal corneal nerve fibres, pinprick hyperalgesia and greater levels of anxiety and depression. Future mechanistic studies of FMS should assess the neuroaxis and peripheral nervous system to delineate the relative contribution to pathological processes and pain.

## Methods

### Participants

Thirty-three patients with FMS were recruited sequentially from musculoskeletal, fibromyalgia services, pain clinics as well as community fibromyalgia patient support groups. Ten healthy volunteers were also recruited as part of the study. Written informed consent was obtained from each participant and the study conduct adhered to good clinical practice guidelines and the tenets of the Declaration of Helsinki (South West—Frenchay Research Ethics Committee REC reference: 20/SW/0138). Inclusion criteria were people aged 18 years or over with a diagnosis of FMS based on current guideline criteria: generalised pain, defined as pain in at least 4 or 5 regions, present for at least 3 months, with either a widespread pain index ≥ 7 and symptom severity scale score ≥ 5 or widespread pain index of 4–6 and symptom severity score ≥ 9^42^, willing and able to provide informed consent. We excluded other causes of neuropathy (including diabetes, prediabetes and rheumatological disorders e.g. Sjorgren’s syndrome, rheumatoid arthritis, mixed connective tissue disorders, etc.) based on a clinical/family history and biochemistry/blood. As a consequence, serum was collected for HbA1c, B_12_, folate, renal profile, liver profile, full blood count, thyroid function tests, immunoglobulins & electrophoresis, and relevant auto-antibodies e.g., anti-CCP, and anti-nuclear antibody with extractable nuclear antigens (ENAs) to exclude other causes for peripheral neuropathy and connective tissue disorders. Any disorder either systemic or localised disease (including severe dry eye) which may cause pathology of corneal nerves were also excluded. Participants with no history of FMS in addition to the above exclusions were recruited as healthy volunteers from friends, carers of FMS participants and visitors and staff of the clinical centre.

### Questionnaires

Participants completed five questionnaires to assess the presence of pain, neuropathic symptoms and the impact of FMS on day-to-day life. The Revised Fibromyalgia Impact Questionnaire (FIQR) was administered to determine the severity of participants symptoms and functional impairment, including physical impairment, ability to work, restfulness, and mood^43^. The Neuropathy Symptom Profile (NSP) was used to assess sensory, autonomic and motor neuropathy symptoms^44^. The Small Fibre Neuropathy Screenings List (SFNSL) evaluated symptoms of small nerve fibre related symptoms^45^. The PainDETECT screening tool evaluated neuropathic pain symptoms^46^. An additional measure of current pain score was assessed using the McGill visual analogue scale (VAS 0–10)^47^.

### Corneal confocal microscopy

Participants underwent corneal examination with the Heidelberg Retina Tomograph 3 with Rostock Cornea Module (Heidelberg Eye Explorer, Heidelberg Engineering GmBH, Heidelberg, Germany) and images of the corneal sub-basal nerve plexus were captured following an established protocol^48^. All experimental protocols were approved by South West—Frenchay Research Ethics Committee. Image selection was masked to the subgroups and average number of images for analysis per participant were 16. Automated analysis was conducted using ACCMetrics software (ACCMetrics: Malik Lab, Imaging Science, University of Manchester)^21^. Three corneal nerve parameters were quantified from each image: (1) corneal nerve fibre density (CNFD) [total number of main nerve fibres per square millimetre of corneal tissue (fibre no/mm^2^)]; (2) corneal nerve branch density (CNBD) [number of branches of all main nerve fibres per square millimetre of corneal tissue (branch no./mm^2^)]; and (3) corneal nerve fibre length (CNFL) [the total length of all main nerve fibres and branches (mm/mm^2^) within the images]^49^.

An abnormal reduction in small nerve fibres was considered CNFL ≤ 14.6 mm/mm^2^^21^. This CNFL cut-off has previously been validated in patients with diabetic neuropathy (AUC values higher for both manual and automated CNFL relative to the other CCM metrics^50^) and was used to divide patients into those with (SFP+) and without (SFP−). To further investigate the relationship between structural small fibre alterations and symptoms associated with small fibre neuropathy, we further subdivided the SPF+ and SPF− patient cohorts into patients without small fibre neuropathy symptoms and those with symptoms compatible with small fibre neuropathy based on SFNSL score (> 48)^45^.

### Quantitative sensory testing

A full QST battery, representing seven tests assessing 13 parameters, was performed on the right hand using the standardized German DFNS testing protocol^23^. Tests for thermal sensation were performed at the beginning of the testing paradigm, prior to mechanical assessments. Thermal tests were performed using the TSA-II NeuroSensory Analyser Medoc, Ltd., Ramat-Yishai, Israel; thermode size 16 × 16 mm. Cold and warm detection thresholds (CDT, WDT), cold and heat pain thresholds (CPT, HPT) as well as thermal sensory limen (TSL) were assessed. Paradoxical heat sensations (PHS) were also recorded. Mechanical detection threshold (MDT) was determined using Von Frey hairs (Opti-hair2-Set, Marstock Nervtest, Germany). Mechanical pain threshold (MPT), mechanical pain sensitivity (MPS) and wind-up ratio (WUR) were all assessed using pinprick stimulators with standardized intensities (8, 16, 32, 64, 128, 256 and 512 mN)^23^. Dynamic mechanic allodynia was assessed using a cotton wisp (exerting a force of 3 mN), a Q-tip (exerting a force of 100 mN) and a soft brush (exerting a force of 200–400 nM), applied in a balanced order and pain ratings recorded. Pressure pain threshold (PPT) was evaluated using a pressure algometer (FDN200, Wagner Instruments, USA) with a blunt contact area of 1 cm^2^ placed on the thenar eminence. Vibration detection threshold (VDT) was recorded using a tuning fork (Rydel Seiffer 64 Hz with fixed weights) placed on the bony styloid process of ulnar^51^. The raw QST data from each test was log transformed and converted into z-scores (with exception of paradoxical heat sensations and dynamic mechanical allodynia) to normalize the data for age, sex and body site tested. Positive z-score values denote a gain in function and negative z-scores denote a loss of function in each of the parameters. Values less than − 1.96 (loss of function) or greater than 1.96 (gain of function) are considered abnormal.

### Conditioned pain modulation

Conditioned pain modulation was used to assess efficiency of diffuse noxious inhibitory control. Pressure pain threshold on the right abductor pollicis brevis was used as the test stimulus. A pressure algometer (FDN200, Wagner Instruments, USA) with a blunt contact area of 1 cm^2^ was placed on the skin above the abductor hallucis muscle on the right hand. Pressure was applied with increasing intensity at a rate of 0.5 kg (50 kPa)/s. The participant indicated as soon as the sensation of pressure changed to an additional painful ‘burning’, ‘stinging’ or ‘aching’ sensation and the value on the algometer recorded. The test was repeated three times with a break of 10 s in between and mean value recorded. Noxious cold was used as the conditioning stimulus, with the left hand of the patient immersed up to the wrist in a water bath of melting ice water for up to 180 s or as long as the participant could tolerate, with a minimum time of 45 s. Pain ratings, using a numerical rating scale of 0–100, were recorded every 15 s. Following removal of the hand from the water bath, the test stimuli were repeated on the right hand (non-submerged) as detailed above. The conditioned pain modulation effect was calculated as the difference (post conditioning stimulus minus pre) in pressure pain thresholds. A positive value indicates efficient conditioned pain modulation.

### Statistical analysis

Statistical analyses were performed using GraphPad Prism statistical software (GraphPad Software Inc, La Jolla, CA, USA). Parametric data were analysed using one-way ANOVA and Tukey’s multiple comparison test to compare means between groups. Results were reported as mean ± standard deviation. For non-parametric data, Kruskal–Wallis and Dunn’s multiple comparison test was used to compare between group means, with results reported as median ± interquartile range. Significance (p) and test statistic F or H was reported for parametric and non-parametric data respectively. Effect size (eta squared η^2^) was reported for values reaching the significance threshold set at < 0.05. MPS results were dichotomised into normal or abnormal and compared with chi-square statistics among four subgroups. Three patients were excluded from the analysis due to missing or technically compromised data.

## Data Availability

The datasets generated and analysed during the current study are available from the corresponding author on reasonable request.

## Abbreviations

CCM: Corneal confocal microscopy
CDT: Cold detection threshold
CNBD: Corneal nerve branch density
CNFD: Corneal nerve fibre density
CNFL: Corneal nerve fibre length
CPT: Cold pain threshold
DFNS: Deutsche Forschungsverbund Neuropathischer Schmerz (German Research Network on Neuropathic Pain)
FIQR: Fibromyalgia impact questionnaire (revised)
HPT: Heat pain threshold
IENFD: Intraepidermal nerve fibre density
MDT: Mechanical detection threshold
MPS: Mechanical pain sensitivity
MPT: Mechanical pain threshold
PPT: Pressure pain threshold
QST: Quantitative sensory testing
SFN: Small fibre neuropathy
SFNSL: Small fibre neuropathy screening list
SFP: Small fibre pathology
SFP−: Without small fibre pathology
SFP+: With small fibre pathology
TSL: Thermal sensory limen
VAS: Visual analogue scale
VDT: Vibration detection threshold
WDT: Warm detection threshold
WUR: Wind up ratio
